# Erythroid Progenitor Cells in Atlantic Salmon (*Salmo salar*) May Be Persistently and Productively Infected with Piscine Orthoreovirus (PRV)

**DOI:** 10.3390/v11090824

**Published:** 2019-09-05

**Authors:** Muhammad Salman Malik, Håvard Bjørgen, Kannimuthu Dhamotharan, Øystein Wessel, Erling Olaf Koppang, Emiliano Di Cicco, Elisabeth F. Hansen, Maria K. Dahle, Espen Rimstad

**Affiliations:** 1 Department of Food Safety and Infection Biology, Faculty of Veterinary Medicine, Norwegian University of Life Sciences, 0454 Oslo, Norway (M.S.M.) (K.D.) (Ø.W.) (E.F.H.); 2 Department of Basic Science and Aquatic Medicine, Faculty of Veterinary Medicine, Norwegian University of Life Sciences, 0454 Oslo, Norway (H.B.) (E.O.K.); 3 Pacific Biological Station, Fisheries and Oceans Canada, Nanaimo, BC V9T 6N7, Canada; 4 Department of Fish Health, Norwegian Veterinary Institute, 0454 Oslo, Norway

**Keywords:** PRV-1, piscine orthoreovirus, persistence, erythroid progenitor cells

## Abstract

Piscine orthoreovirus (PRV-1) can cause heart and skeletal muscle inflammation (HSMI) in farmed Atlantic salmon (*Salmo salar*). The virus targets erythrocytes in the acute peak phase, followed by cardiomyocytes, before the infection subsides into persistence. The persistent phase is characterized by high level of viral RNA, but low level of viral protein. The origin and nature of persistent PRV-1 are not clear. Here, we analyzed for viral persistence and activity in various tissues and cell types in experimentally infected Atlantic salmon. Plasma contained PRV-1 genomic dsRNA throughout an 18-week long infection trial, indicating that viral particles are continuously produced and released. The highest level of PRV-1 RNA in the persistent phase was found in kidney. The level of PRV-1 ssRNA transcripts in kidney was significantly higher than that of blood cells in the persistent phase. In-situ hybridization assays confirmed that PRV-1 RNA was present in erythroid progenitor cells, erythrocytes, macrophages, melano-macrophages and in some additional un-characterized cells in kidney. These results show that PRV-1 establishes a productive, persistent infection in Atlantic salmon and that erythrocyte progenitor cells are PRV target cells.

## 1. Introduction

Marine farming of Atlantic salmon (*Salmo salar*) has become a major industry. Infectious diseases pose a significant threat for intensive aquaculture, and pathogens produced in dense farmed populations may be extensively released and be a threat for wild fauna [1]. Piscine orthoreovirus (PRV) is a virus with ubiquitous presence in farmed Atlantic salmon in the final half of the marine grow out phase [2]. PRV can induce heart and skeletal muscle inflammation (HSMI) in Atlantic salmon [3,4], where the major pathological changes are moderate to severe endo-, myo- and epicarditis and red skeletal myositis and myonecrosis [5]. However, PRV can be present in Atlantic salmon without HSMI lesions, indicating that factors related to virus strains, host, and environment including management influence the outcome of the infection [5,6,7,8]. HSMI causes low to moderate mortality (0–20%), but the number of HSMI outbreaks is high in some areas, leading to a significant effect on the Atlantic salmon aquaculture industry.

PRV belongs to the family Reoviridae, genus Orthoreovirus, which has a 10-segmented double stranded RNA (dsRNA) genome enclosed in a double-layered protein capsid [9]. The genomic segments are divided into three groups; long (L1-L3), medium (M1-M3) and small (S1-S4). Currently there are three identified genotypes of PRV; some forms of PRV-1 causes HSMI in Atlantic salmon [4]; PRV-2 causes erythrocytic inclusion body syndrome (EIBS) in coho salmon (*Oncorhynchus kisutch*) [10], and PRV-3 causes heart inflammation in Rainbow trout (*Oncorhynchus mykiss*) [11]. 

A number of studies addressing of PRV-1 have focused on the acute phase of infection, which is characterized by infection of erythrocytes and cardiomyocytes, leading to the development of HSMI [12]. Piscine erythrocytes are nucleated and particularly young salmonid erythrocytes contain the transcriptional and translational machinery enabling virus replication [13]. In an experimental challenge study of EIBS in coho salmon, inclusions appeared only in immature erythrocytes at an early stage in the disease development [14]. The acute phase of PRV-1 infection in Atlantic salmon erythrocytes occurs 4-6 weeks after virus exposure and lasts for approximately 1-2 weeks, after which the virus protein load falls radically in blood cells, and the virus infection transfers into persistence [15]. The PRV structural and non-structural proteins, apart from a fragment of the structural protein µ1, are below detection level after the acute phase [15]. On the other hand, the level of viral RNA in blood cells does not drop accordingly; which indicates continuous viral transcription with partial arrest of viral translation. Generally, farmed Atlantic salmon do not eliminate PRV-1, since viral RNA can be detected in different tissues for at least 36 week post infection in experimental trials (end of experiment) [2], and in blood for more than a year after challenge [16]. PRV-1 strains that cause HSMI induce an innate antiviral immune response in erythrocytes in the acute phase of the infection [17], while infection with PRV-1 strains from BC, Canada associated with lack of or mild clinical signs and no elevated mortality and only a low antiviral transcriptional response in the host [8,18]. However, establishment of persistent infection, requires viral evasion of the innate immune response, i.e., viral dsRNA must be shielded from dsRNA recognizing receptors [19]. Studies of the mammalian orthoreovirus (MRV) have shown that genomic dsRNA is not exposed in the cytoplasm, as viral mRNA transcription occurs within core particles [20]. New positive ssRNA strands are encapsidated before they template the synthesis of negative strands to form the dsRNA genome [21]. Less information is available for PRV replication, since all genotypes have resisted cultivation in fish cell lines, and most studies have been performed in experimentally infected fish.

Persistent infections of PRV-1 in farmed Atlantic salmon represent a formidable reservoir of virus, with estimated more than 400 million infected individuals per year in Norway alone. An experimental trial indicated that fish persistently infected with PRV-1 do not continuously shed the virus, since PRV-1 was not transmitted to sentinel fish from persistently infected shedders at 59 weeks post challenge [16]. On the other hand, farmed fish may be immunosuppressed due to crowding, transportation, handling and various treatments, possibly allowing the virus production to proceed. For example, fish persistently infected with infectious pancreatic necrosis virus (IPNV), another dsRNA virus, have been found to be intermittent virus shedders [22]. However, a recent study found that the prevalence of PRV in farmed escapees (86%) was significantly higher than in wild salmon (8%), and did not find association between salmon farming and prevalence of PRV infection in wild salmon [23].

In this study, we characterized the viral kinetics during the acute and persistent phase of PRV-1 infection in Atlantic salmon. PRV-1 establishes a persistent, low-activity, but productive infection in Atlantic salmon. Using *in situ* hybridization techniques for cell-specific detection of PRV, PRV-1 persistence was mapped to the erythropoietic tissue of kidney, particularly to erythroid progenitor cells, macrophages, melano-macrophages, and erythrocytes.

## 2. Materials and Methods 

### 2.1. Experimental Challenge 

Atlantic salmon smolts, approximately of 200 g size at onset of experiment, were reared in tanks at 40 kg/m^3^, supplied with particle filtered and UV treated seawater at 12 °C ± 1 °C, 34 ‰ salinity and 24-hour daylight regime. The fish originated from broodstock found negative by RT-qPCR for infectious salmon anemia virus (ISAV), salmonid alphavirus (SAV), infectious pancreatic necrosis virus (IPNV) and PRV. The study population was tested and found negative for these agents before recruitment to the experiment. The PRV-1 NOR2012 isolate was used as challenge material, an isolate that originated from an HSMI outbreak in 2012 and that has been repeatedly used in experimental infections reproducing HSMI [4]. The inoculum consisted of pelleted blood cells diluted in PBS. It was administered by intraperitoneal (i.p.) injection using 0.1 mL per fish to ensure a defined time point of infection (*n* = 42). Control group of uninfected fish (*n* = 42) were kept in a separate tank. The fish were anesthetized by bath immersion in benzocaine chloride (2–5 min, 0.5 g/10 L) prior to handling, and euthanized using overdose of benzocaine chloride (1 g/5 L). Sampling (*n* = 6) was done every third week by harvesting heart, spleen, kidney, skeletal muscle and blood cells in RNAlater™ (Thermo Fischer Scientific, Waltham, MA, USA) and in formalin until termination of the study at 18 weeks post challenge (wpc). Plasma was collected from the blood samples. The sampling intervals reflect a focus on the persistent phase and not the viral peak or eventual development of histopathological lesions typical of HSMI. 

### 2.2. Ethics Statement

An experimental challenge with PRV-1 was performed at the VESO Vikan research facility (Namsos, Norway), in compliance with the regulatory requirements by Norwegian Food Safety Authority, EU Council Directive 2004/10/EC and Guidelines to Good Manufacturing Practice by European Commission Directives 2003/94/EC and 91/412/EC. The Norwegian Food Safety Authority (NFDA) according to the European Union Directive 2010/63/EU for animal experiments approved the experiment (use protocol V3740). 

### 2.3. RNA Isolation

Total RNA was isolated from pelleted blood cells (20 µL), spleen and kidney samples (25 mg) by using QIAzol Lysis Reagent (Qiagen, Hilden, Germany), TissueLyser II (Qiagen) with 5 mm steel beads for 2 × 5 min at 25 Hz followed by chloroform addition and collection of the aqueous phase. RNeasy QIAcube Kit (Qiagen) was used for automated RNA isolation of the aqueous phase as described by manufacturer. RNA was quantified in a NanoDrop ND-100 spectrophotometer (Thermo Fisher Scientific, Waltham, MA, USA). For cell free samples (plasma), 10 µL plasma was diluted in 130 µL PBS and QIAamp Viral RNA Mini QIAcube Kit (Qiagen) used according to the manufacturer instructions. Isolated RNA was eluted in 60 µL RNase-free water and stored at −80 °C until further use.

### 2.4. RT-qPCR

For transcription analysis of the individual PRV segments, cDNA was synthesized from 1 µg RNA of spleen tissue and blood cells using Quantitect Reverse Transcription Kit (Qiagen) with genomic DNA elimination (Qiagen) following the manufacturer’s instructions with prior denaturation of RNA at 95 °C for 5 min. Quantitative PCR was performed using 15 ng cDNA input in a total reaction volume of 12 µL and Maxima SYBR Green/ROX qPCR Master Mix (2X)-K0253. qPCRs were run with initial denaturation for 10 min/95 °C and 40 cycles of 15 sec/95 °C, 30 sec/60 °C and 30 sec/72 °C. Cut-off value was set to Ct 34. Specificity of assays were confirmed by melting point analysis, and all samples were run on the same plate with positive and no template controls (NTC). Elongation factor (EF1α) was used as reference gene and its expression in spleen and blood cells for the individual fish is showed in Figure S1.

For PRV S1 segment, one-step RT-qPCR assay was performed for blood cells and kidney samples using Qiagen OneStep kit (Qiagen) with 100 ng (5 µL of 20 ng/µL) RNA per reaction, or purified RNA from 5 μL plasma, in duplicates of 12.5 µL total reaction volume. RNA was used both with and without a prior denaturation step, i.e pre-heating at 95 °C of the template to evaluate ratio between genomic dsRNA and single stranded transcripts [8]. Cycling parameters were 30 min/50 °C, 15 min/95 °C, 40 cycles of 15 sec/94 °C, 30 sec/60 °C and 30 sec/72 °C. Samples were run in duplicates and cut off value was set to Ct 35 [3]. Analyses were based on mean Ct-value of six fish per group per sampling. Sequences of probes and primers, and specific concentrations are listed in Table 1. Primer sequences were designed by software MEGA version 7.0 and open source primer-3 applications.

### 2.5. Data Analysis

RT-qPCR data was analyzed and graphically laid out with Graphpad Prism version 8.1.1 (Graphpad Software Inc., La Jolla, CA, USA). Statistical analysis was performed to measure PRV genomic segment expression, ratio of PRV genomic segments and viral transcripts in tissue and ratio of ssRNA viral transcripts level in blood cells and kidney by applying Wilcoxon matched-pairs signed rank test, and *p*-values of *p* ≤ 0.05 were considered as significant.

### 2.6. Western Blotting

Heparinized blood cell pellets from the experimental PRV-1 challenged Atlantic salmon were used for virus protein expression analysis in western blotting (WB). Heparinized blood from PRV-1 infected and uninfected fish from a previous challenge trial were used as positive and negative controls, respectively [3]. For each sample, 15 µL blood pellet was added to Nonidet-P40 lysis buffer containing complete ultra mini protease inhibitor cocktail (1:5) (Roche, Mannheim, Germany) and incubated for 30 min on ice. After centrifugation at 5000× *g* for 5 min, the supernatant was diluted with DEPC treated water (1:5) and mixed with XT sample buffer and XT reducing agent (Bio-Rad, Hercules, CA, USA) and boiled at 95 °C for 5 min. PRV proteins were separated by sodium dodecyl sulfate-polyacrylamide gel electrophoresis (SDS-PAGE), using 4–12% criterion XT bis-tris gel. TransBlot Turbo (Bio-Rad) for 20 min at 15 V transferred proteins to polyvinylidene fluoride (PVDF) membranes (Bio-Rad). The membranes were incubated at 4 °C overnight using polyclonal rabbit sera anti-σ1 and anti-μ1C diluted 1:500; [24]. Rabbit anti-actin (Sigma-Aldrich, St. Louis, MO, USA) was used to standardize the amount of protein added to the blots. Horseradish peroxidase (HRP)-conjugated anti-rabbit IgG (1:20,000) (Amersham, GE Healthcare, Buchinghamshire, UK) was used as secondary antibody. For immune detection, Clarity Western ECL Substrate kit (Bio-Rad) was used along with Precision Plus Protein™ (Bio-Rad) as molecular weight ladder. Images were developed by ChemiDoc XRS+ System and ImageOne software (Bio-Rad).

### 2.7. In-Situ Hybridization (ISH)

RNAscope^®^ (RED) 2.5 HD Detection Kit (Advanced Cell Diagnostic, Newark, CA, USA) was used for RNA-ISH, following the instructions of the manufacturer. Paraffin embedded tissue sections (5 µm) of spleen, kidney and heart from PRV-challenged fish with lowest Ct values from each sampling were dewaxed at 60 °C for 90 min in ACD HybEZ™ II followed by hydrogen peroxide for 10 min incubation at room temperature. Samples were boiled in RNAscope target antigen retrieval reagent for 15 min and each section was incubated with RNAscope protease plus at 40 °C for 15min in HybEZ™ oven. Each section was hybridized by RNAscope probe (Table 2) designed against PRV-1 genome segment L3 (Advanced Cell Diagnostics catalog number-537451), that encodes inner capsid protein (helicase) for 2 hrs at room temperature. Probe targeting Peptidylpropyl Isomerase B (PPIB) in Atlantic salmon (Advanced Cell Diagnostics, catalog number-494421) was used reference target gene expression to test for RNA integrity in the samples. As negative control, probe-DapB (Advanced Cell Diagnostics catalog number-310043) were used to evaluate cross reactivity (Figure S2). Fast Red chromogenic substrate was used for detection of signals amplified following manufacturer’s instructions. Counterstaining was done with 50% Gill’s hematoxylin solution and mounted with EcoMount (BioCare Medical, Pacheco, CA, USA). Imaging was performed by bright field microscopy (Carl Zeiss Light Microscopy System with Axio Imager 2 (Carl Zeiss AG, Oberkochen, Germany). Fish injected i.m. (*n* = 42) with heat inactivated (85 °C, 20 min) PRV-1 infected erythrocytes were used as control fish (Figure S5).

### 2.8. Duplex In-Situ Hybridization

RNAscope^®^ 2.5 HD Duplex Detection Kit-Chromogenic for simultaneous detection of two RNA targets (Advanced Cell Diagnostics) was used for kidney and spleen samples. Probes for PRV-1 segment L3 was combined with probes for macrophage colony stimulating factor (MCSFR) (Advanced Cell Diagnostics catalog number-557811) or erythropoietin receptor (EPOR) (Advanced Cell Diagnostics catalog number-561741) (Table 2). Additional amplification steps were applied (Amp1-Amp10) for the duplex assay according to manufacturer’s instructions. Signals were detected by using Green substrate specific for HRP conjugated probes and Fast Red for Alkaline phosphatase (AP) conjugated probes. Each slide was counter stained with 50% Gill’s hematoxylin staining solution and mounted with VectaMount (Cat. No: H-5000, Vector Labs, Burlingame, CA, USA).

## 3. Results

### 3.1. PRV-1 Segments Have Similar Expression Pattern

**Figure 1:**
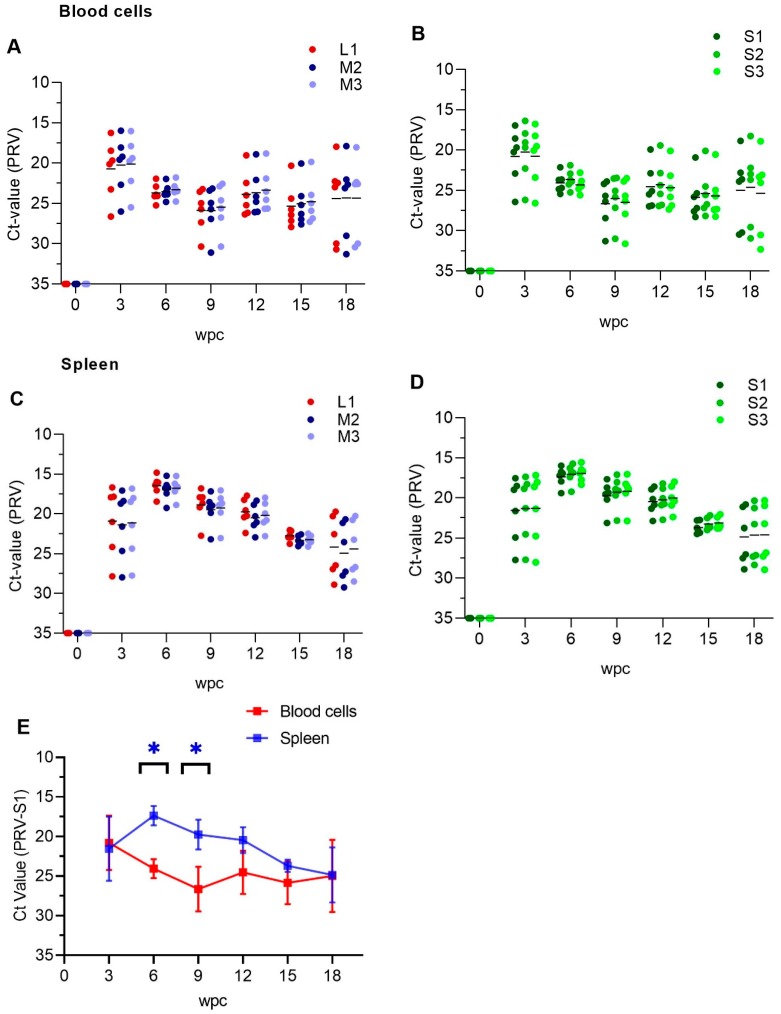
Expression analysis of PRV-1 segments and transcripts. qPCR detection of segment L1, M2, M3, S1-S3 and corresponding transcripts in blood cells (**A**,**B**) and spleen (**C**,**D**) from Atlantic salmon. Individual fish shown as dots, and mean Ct value-PRV as lines. n = 6 for each point. (**E**) S1 as a representative for PRV genomic segments expression between blood cells and spleen. Paired analysis was performed by non-parametric Wilcoxon matched pairs signed rank test (*p* < 0.05), asterisk (*) indicates significantly different group. wpc = weeks post challenge.

The segment expression levels, judged through Ct values, were very similar as assessed in a time course study of PRV-1 infection (0–18 weeks). RNA levels of various PRV-1 segments in blood and spleen were mapped in order to investigate potential differential expression pattern of the segments in the acute and persistent phases of infection. The analysis targeted the genomic segments L1, M2, M3, S1, S2 and S3; and individual fish with a low number of L and M segment copies, also had low number of S segment copies (Figure 1). In blood cells, the amount of PRV-1 RNA levelled out from 6 wpc towards the end of the study (18 wpc) (Figure 1A,B). In spleen, peak PRV RNA load for all segments was reached at 6 wpc, followed by a gradual reduction in viral RNA (Figure 1C,D). Figure 1E shows the difference in the PRV-1 RNA loads in blood, i.e., levelling out from 6 wpc, versus that of spleen, i.e., a more gradual reduction. PRV segment expression levels were not significant different. PRV-1 was not cleared from any of the sampled fish, but at 18 weeks the PRV RNA level varied more between individual fish compared to the preceding samplings (Figure 1).

### 3.2. Viral Genomic RNA Versus Viral Transcripts

**Figure 2:**
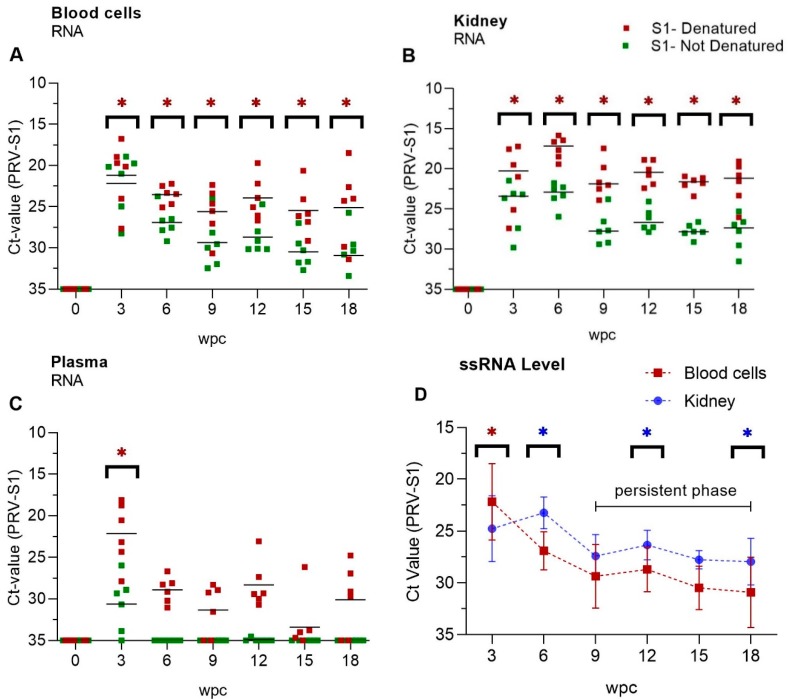
PRV-1 dsRNA and ssRNA levels. Mean Ct-values of RT-qPCR (PRV-S1) for (**A**) Blood cells, (**B**) Kidney and (**C**) Plasma. RNA was pre-heated (red dots) or not (green dots), indicating presence of viral dsRNA plus ssRNA transcripts or ssRNA PRV transcripts only, respectively. *n* = 6 for each time point. Statistical analysis between denaturated and not-denaturated samples from the same RNA was performed by non-parametric, Wilcoxon matched pairs signed rank test. Asterisk shows significantly high level (* *p* < 0.05) (**D**) Paired analysis of ssRNA Ct-values (Table S1) of kidney compared to blood cells in persistent phase (9 wpc -18 wpc). Each dot shows mean Ct-value at each time point. Statistical analysis was performed by non-parametric, Wilcoxon matched pairs signed rank test (*p* < 0.05), asterisk (red/blue) indicates significantly different group.). wpc = weeks post challenge.

Genomic dsRNA levels were higher in both blood cells and kidney than PRV transcripts at each time point of the experiment (Figure 2A,B). Isolated RNA with or without prior denaturation before cDNA synthesis was used in qPCR to estimate the ratio between viral genomic dsRNA and ssRNA transcripts, where the dsRNA will make up the difference between denatured and non-denatured RNA (Table S1). At the peak of infection at 3 wpc, ssRNA viral transcripts levels were high in blood cells and ΔCt value between denatured and non-denatured was 1, i.e., ssRNA made up approximately 50% of the viral RNA (Figure 2A). For the samples from 6 wpc onward the ΔCt between denatured and non-denatured RNA was on average −4 for blood cells. It indicated that the viral genomic dsRNA level was approximately 16 fold higher, or that ssRNA made up approximately 6% of the viral RNA in blood cells (Figure 2A, Table S1A). Paired data analysis showed significantly high levels of ssRNA PRV transcripts in blood cells during acute phase i.e 3wpc, and in kidney during persistent phase (9 wpc–18 wpc) of infection, when compared to each other (Figure 2D). The ssRNA level in kidney was higher during persistent phase indicating active transcription and viral activity in this organ. In plasma, viral ssRNA transcripts were detectable at the viral peak at 3 wpc, but not thereafter (Figure 2C, Table S1C), while PRV-1 genomic dsRNA was present in plasma throughout experiment (Figure 2C). However, at 9, 15 and 18 wpc the dsRNA could not be detected in 2-3 out of six fish.

### 3.3. Low Level of PRV-1 Protein Synthesis in the Persistent Phase

**Figure 3:**
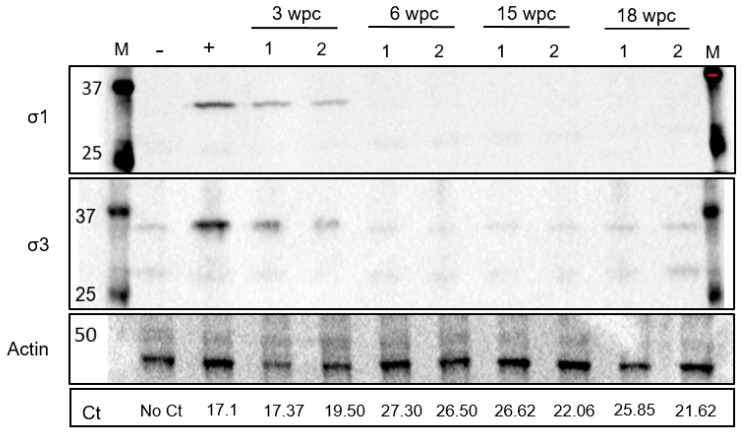
Low levels of PRV proteins in the persistent phase. Detection of outer PRV-1 capsid proteins σ1 and σ3 in blood cells from 3–18 weeks post challenge (wpc) by western blotting. Ct values in lower row. Actin (42 kDa) was used as a protein load control. Blood cells from uninfected fish used as negative control. Positive control sample from a fish having Ct value of 17.1 and significant σ1 protein level [15].

Western blotting did not detect expression of PRV σ1 and σ3 proteins in blood cells in the persistent phase. Samples from fish with high loads of viral RNA, collected from the acute phase of infection at 3 wpc and 6 wpc, were compared to samples from the persistent phase of infection at 15 wpc and 18 wpc (two fish per time point). σ1 specific bands (34.6 kDa) were only detected in samples from 3 wpc. Similarly σ3 specific bands (approximately 35 kDa) were also observed, but bands were not stronger than negative control thereafter (Figure 3).

### 3.4. Tissue Localization of PRV-1 in the Acute Phase

*In situ* hybridization targeting PRV was performed on 3 samples from individual fish from each time point, 3 wpc and 6 wpc representing the acute and early persistent phase, respectively, and 9 wpc and 18 wpc representing the persistent phase. PRV demonstrated of tissue specific localization in heart, spleen and kidney.

**Figure 4:**
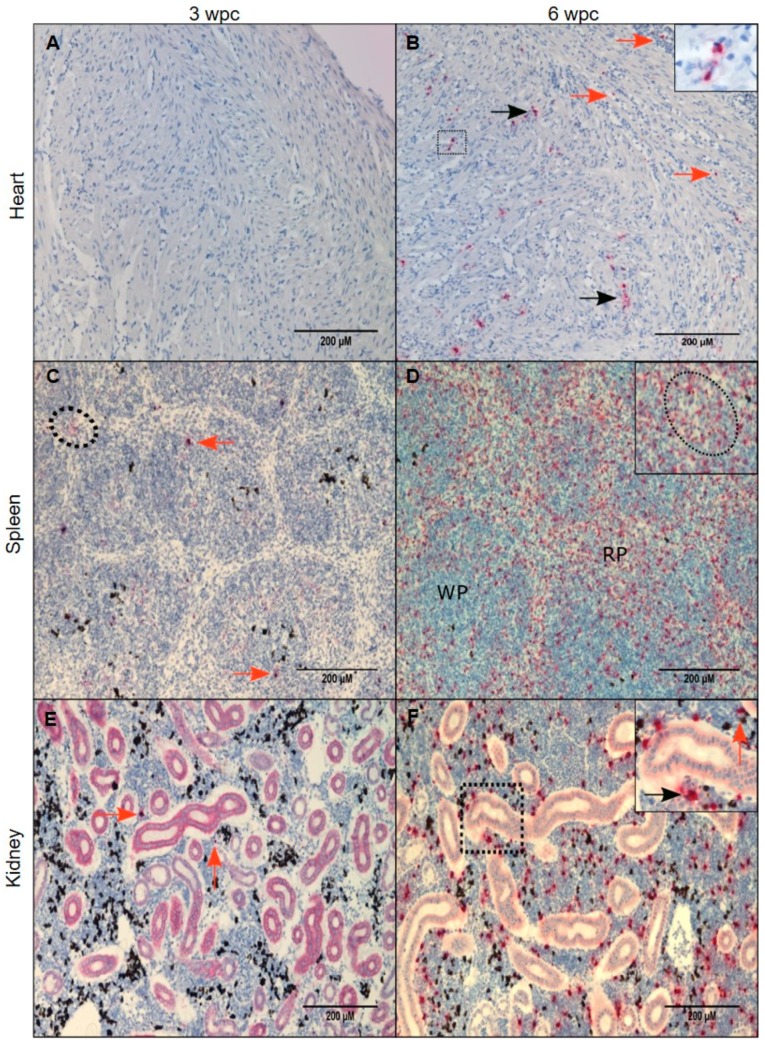
PRV-1 localization in the acute phase. PRV-1 localization shown by *in situ* hybridization (red) at 3 and 6 wpc. Heart (**A**,**B**): A, (3 wpc), no PRV detection. B, (6 wpc), PRV positive cardiomyocytes in compact layer (orange arrows) and a few positive erythrocytes or infiltrating macrophage-like cells were present in the epicardium and scattered PRV staining in the spongy layer of the heart ventricle (black arrows). Spleen (**C**,**D**): C, (3 wpc), few PRV-1 positive RBCs (dotted circle) and macrophage-like cells (orange arrows). D (6 wpc) shows a large number of positively stained cells in the red pulp (RP) area. Dotted circle in magnified version in upper right corner shows cluster of cells with erythrocyte- and macrophage-like morphology. Kidney (**E**,**F**): E, (3 wpc) shows a few PRV-1 positive cells (orange arrow), primarily macrophage-like cells. F, (6 wpc), a high number of PRV-1 positive macrophage-like cells (black arrow) along with melano-macrophages (orange arrow) were found in peritubular regions of the kidney. Scale bar = 200 µm.

In heart, PRV-1 was not detected at 3 wpc (Figure 4A). At 6 wpc, numerous cardiomyocytes in the spongy layer (*stratum spongiosum*) of the heart ventricle showed abundant viral staining, whereas a few positive cells in the inner part of compact layer (*stratum compactum*) (Figure 4B) and a few positive erythrocyte-like cells or infiltrating macrophage-like cells were present in the epicardium.

In spleen at 3 wpc, some erythrocytes, melano-macrophages and cells with macrophage-like morphology (“macrophage-like cells”) were positively stained (Figure 4C). At 6 wpc PRV-1 was localized in large number of erythrocyte-like cells in the spleen. These cells had aberrant shape or signs of damage. Red pulp contained primarily PRV-1 positive cells. Clusters of PRV-1 positive macrophage- and erythrocyte-like cells were scattered throughout the tissue (Figure 4D).

In kidney, primarily cells morphologically assessed as macrophage-like and in melano-macrophages stained PRV-1 positive. At 3 wpc, only a few positive cells were identified (Figure 4E). At 6 wpc, several PRV-1 positive macrophage-like cells and melano-macrophages were found in the peritubular regions of the kidney (Figure 4F).

### 3.5. Tissue Localization of PRV-1 in the Persistent Phase

**Figure 5:**
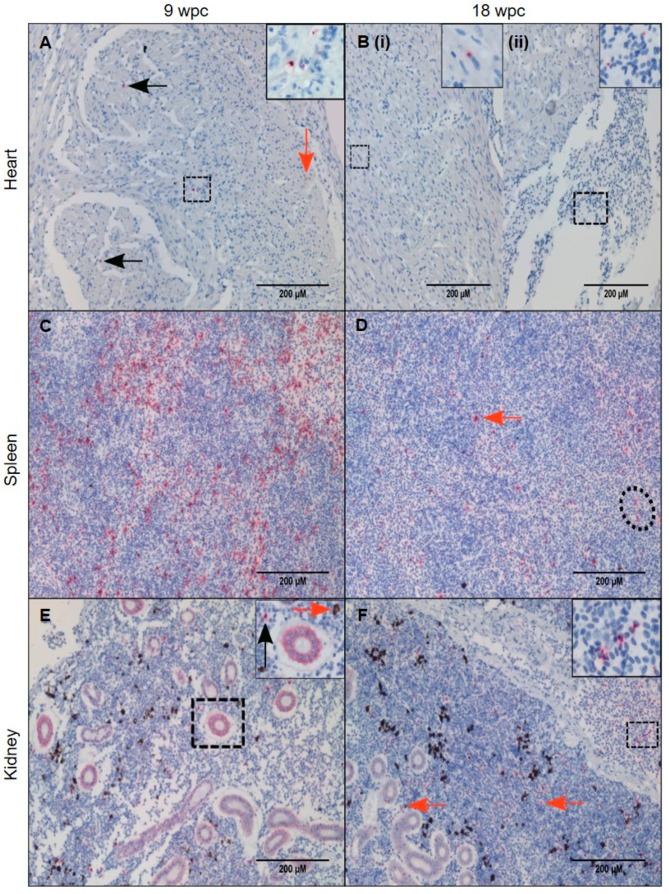
PRV-1 localization during the persistent phase. PRV-1 localization shown by *in situ* hybridization (red) at 9 and 18 wpc. Heart (**A**,**B**): A, (9 wpc) A small number of cells in the compact layer stained positive for PRV-1 (orange arrow), and similarly faintly in a few macrophage-like cells in the in the luminal space of the spongy layer (black arrows) and some circulatory cells, near blood vessel (dotted square and magnified image). B, (18 wpc) Very few cells were positive for PRV-1 with the exception of (i) some positive cardiomyocytes in the spongy layer (dotted square) or (ii) peripheral blood in the heart (dotted square). Spleen (**C**,**D**): C, (9 wpc) numerous PRV-1 positive cells in red pulp. D, (18 wpc) A few PRV-1 positive macrophage-like cells (orange arrow) and erythrocyte like cells (dotted circle). Kidney (**E**,**F**): E, (9 wpc) PRV-1 present in macrophage-like cells (black arrow) and melano-macrophages (orange arrow). F, (18 wpc) PRV-1 positive erythrocytes and macrophage-like cells throughout the section (orange arrows) and in blood vessels (magnified picture). Scale bar = 200 µm.

PRV-1 gradually cleared from the heart in the persistent phase. Few PRV-1 positive cells were visible in the *stratum compactum* at 9 wpc and 12 wpc, as some positive cardiomyocytes were still detected (Figure 5A and Figure S4). 

Moreover, peripheral blood in the heart contained PRV-1 positive circulatory cells (Figure 5B). Putative PRV staining was very low in the heart at 18 wpc compared to spleen and kidney. In spleen, the number of PRV-1 positive cells gradually dropped during persistence. At 9 wpc there were still a large number of positive cells in the red pulp, while at 18 wpc the number of positive cells, i.e., macrophage-like cells and damaged erythrocytes, was moderate (Figure 5C,D). In kidney, there was also a decrease in virus positive cells over time, but positive macrophage-like cells and erythrocytes in the blood vessels was consistent during the persistent phase (Figure 5E,F). Many PRV-1 positive cells were present in the hematopoietic portion of the kidney (Figure 5E,F).

### 3.6. Characterization of the PRV-1 Infected Cell Populations in Kidney and Spleen

**Figure 6:**
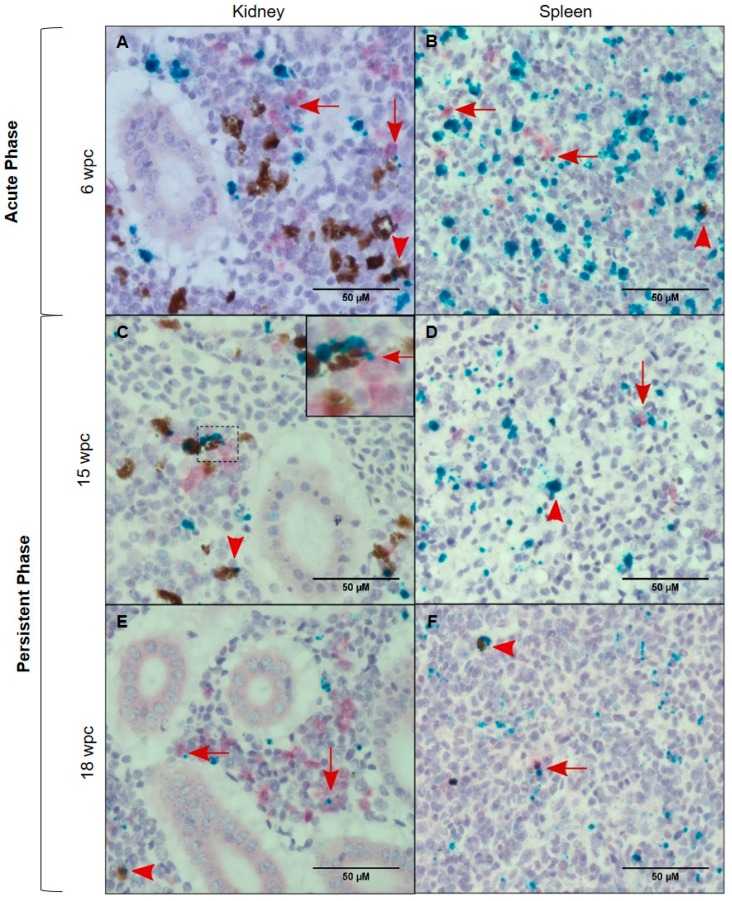
PRV-1 (green) localization in macrophages and melano-macrophages. Duplex *in situ* hybridization against PRV-1 and macrophage colony stimulating factor (MCSFR) (red). Kidney (**A**,**C**,**E**) and Spleen (**B**,**D**,**F**), showing PRV positive macrophages (red arrows) and melano-macrophages (red arrowhead) at 6 wpc, 15 wpc and 18 wpc. The use of red dye in salmon kidney sections in duplex *in situ* gave diffuse staining of positive cells (no background staining). The diffuse staining was different from the punctate staining seen in single *in situ*, or the use of green dye in duplex in situ. PRV-1 is also positive in other cell types in the sections. Scale bar = 50 µm.

*In situ* hybridization against macrophage colony stimulating factor (MCSFR) defined macrophages in the PRV-1 infected population. Co-localization of PRV-1 and MCSFR transcripts in kidney and spleen revealed a few double stained macrophages and melano-macrophages in both the peak phase (6 wpc) and persistent phase of infection (15 wpc and 18 wpc) (Figure 6). 

The red dye defining MCSFR mRNA expression, presented as diffuse staining, which was different from the punctuated staining of green dye. However, the background (Figure 6C,E), was free of the diffuse staining, demonstrating the specificity of the MCSFR staining. Similar diffuse staining with the red dye when applied no kidney and spleen tissue of Atlantic salmon has been observed earlier [25]. PRV-1 localized to some MCSFR positive macrophages; however, this was not the dominating cell type that stained positive for virus. In spleen, the expression of MCSFR dropped after the peak phase, but a few MCSFR positive macrophages, melano-macrophages, erythrocytes and other cells were PRV-1 positive.

**Figure 7:**
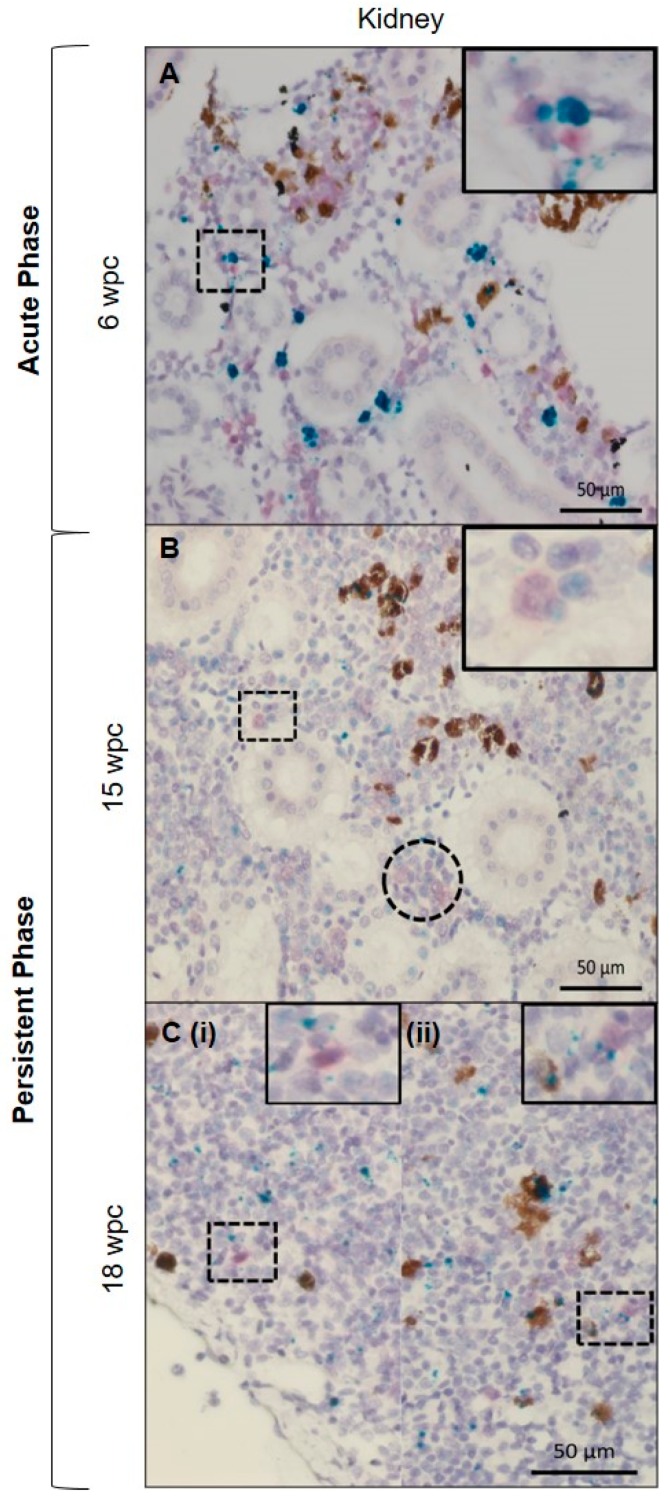
PRV-1 localization in hematopoietic cells. Duplex *in situ* hybridization against PRV-1 (green) and erythropoietin receptor (EPOR) (red). Kidney (**A**) Acute phase, dually stained PRV/EPOR positive cells in hematopoietic tissue in the magnified picture. (**B**,**C**) Persistent phase, dually stained EPOR-PRV1 positive cells in hematopoietic tissue scattered around in the kidney (magnified pictures). Cluster of partially stained positive erythroid cells at 15 wpc (dotted circle). The use of red dye in salmon kidney sections in duplex *in situ* gave diffuse staining of positive cells (no background staining). The diffuse staining was different from the punctate staining seen in single in situ, or the use of green dye in duplex *in situ*. Scale bar = 50 µm.

*In situ* hybridization against erythropoietin receptor (EPOR) defined erythroid progenitor cells in the PRV-1 infected population. Co-staining of EPOR transcripts and PRV-1 in kidney detected some double positive cells (Figure 7). The red dye designating EPOR also gave a diffuse staining, but there was no diffuse staining of the background, demonstrating the specificity of the EPOR staining (Figure 7, inserts). These cells were mainly located in the haematopoietic tissue of the kidney in both the acute and persistent phase. Although both MCSFR and EPOR positive cells were PRV infected, they were not the only infected cell types, as other cell types in kidney and spleen, morphologically resembles macrophage-like, but not staining positive for MCSFR or EPOR, also stained positive for PRV-1.

## 4. Discussion

PRV-1 causes an acute infection of Atlantic salmon erythrocytes, and during the peak of the infection, the virus also infects cardiomyocytes [18,26]. A few weeks later, the pathological changes characteristic for HSMI develop in the myocardium, and subsequently heal. After the heart lesions have healed, PRV infection becomes persistent. In this study, we focused on the kinetics and cell tropism of PRV-1 in the persistent phase of the infection. Since PRV-1 infects erythrocytes also during persistence [16], the virus can be detected by RT-qPCR in any vascularized organ. However, the kidney consistently contained proportionally high levels of PRV transcripts compared to blood cells in the persistent phase, and thus represent the major reservoir of infected cells and potential site of virus production during persistence.

The PRV-1 RNA level in the blood cells stayed moderately stable in the persistent phase while the PRV-1 RNA level gradually dropped in spleen. It was only minor differences in the expression level of the different genomic segments. In the persistent phase, the dominating target was genomic dsRNA and the viral ssRNA transcripts only made up approximately 6% of total RNA. In a recent study of a Canadian PRV-1 isolate, the viral ssRNA represented 0.1–0.7% of the total PRV-1 RNA load in the persistent phase [8]. In the acute phase, i.e., at 3 wpc, the ssRNA fraction constituted approximately 50% of total viral RNA in blood cells, indicating a highly active viral transcription during early infection.

In contrast to PRV-1 RNA, PRV-1 proteins were detectable in blood cells only in the acute phase, in line with earlier findings [15]. The limited virus protein expression after the primary acute infection phase contrasts the continuous high level of PRV-1 RNA. For mammalian reoviruses, the virus protein expression is halted as a result of a partial block in the translational activity of the cell [27]. This effect is caused by the innate antiviral response, and linked to phosphorylation of the alpha subunit of eukaryotic translation initiation factor 2 (eIF2α), executed by the viral dsRNA activated protein kinase (PKR) [28,29]. This can lead to a translationally inactive state of the cell [30].

Viral dsRNA, i.e., genomic viral RNA indicating the presence of intact viral particles, was detected in plasma at all phases of infection. Although the dsRNA level of some individual plasma samples at 9, 15 and 18 wpc were below the detection limit, the average level of viral dsRNA in plasma of the sampled groups was remarkably stable at 9 wpc and 18 wpc. This indicates a continuous virus production and shedding into the circulation. Previously, PRV-1 has been found at high levels in plasma in the acute phase, simultaneous with high viral loads in the blood cells [12]. Our findings of virus in plasma, both in the acute phase and in the persistent phase oppose the previous results from an experiment where two PRV-1 isolates from Canada were administrated to Atlantic salmon. In that particular study, virus was not detected in plasma at any sampling point [8]. However, there is a marked phenotypic difference between the PRV-1 strains used in these experiments. In our experiment, we used the PRV-1 NOR-2012 isolate, which originates from a field HSMI outbreak, and has repeatedly been used in experimental infections that reproduce HSMI [4,17,31,32]. On the other hand, in laboratory-based studies in farmed Atlantic salmon with Canadian PRV-1 isolates are not associated with HSMI-like lesions [16], while in field, cardiac inflammation and mild clinical signs have been reported in one farm [18]. This could suggest a relationship between the ability to produce plasma viremia and induction of HSMI. For the Canadian PRV-1 isolates, the lack of detectable virus in plasma [8] could indicate that the PRV-1 infection of erythrocytes does not primarily take place in blood. If erythroid progenitor cells continuously generate new PRV-1 infected erythrocytes, this would be an ideal long-term reservoir for PRV. Salmonid erythrocytes, like all non-mammalian vertebrate erythrocytes, are cells with nucleus and organelles, and young salmonid erythrocytes are more transcriptionally active than older cells [13]. In line with this, an experimental challenge with EIBS in coho salmon, performed two decades before PRV-2 was described as the etiological cause of EIBS in coho [10], found viral inclusions preferably in young erythrocytes early in the course of the disease [14]. However, in ex vivo systems erythrocytes are susceptible to infection with PRV-1, or at least the HSMI inducing variants of PRV-1 [12].

The salmon immune response is not able to clear the PRV-1 infection, and does not stop PRV-1 from circulating in plasma. However, this does not imply a continuous shedding of infectious virus to the external environment. For the Canadian PRV-1 isolates that do not induce plasma viremia; sentinel fish were not infected when placed in the tank with persistently infected fish, 41 weeks after initial exposure [16].

IgM is the dominating Ig class in salmonid plasma, and virus neutralizing activities of plasma, designated to IgM, has been demonstrated for several viruses [33]. Formation of PRV-specific plasma IgM after infection has been demonstrated, and the peak antibody production corresponded in time with a decrease in myocardial inflammation [34]. Lack of functional antibodies has been reported for infectious pancreatic necrotic virus (IPNV) both in acute and persistent infections, with moderate presence of neutralizing antibodies only in fish with low degree of clinical signs [35]. Splenic ellipsoids are specialized to remove any immune complexes from salmon plasma [36], but the low level of virus in spleen in this phase, indicate that eventual virus-IgM complexes are not readily removed in the persistent phase. Just after the peak phase of infection in blood cells, PRV-1 RNA loads in spleen exceeded levels in blood cells, in line with earlier findings [12]. This corresponded to the *in situ* observations that most of the PRV-1 positive cells were trapped in red pulp of the spleen in the acute phase. Viral RNA loads in spleen gradually dropped after the peak infection phase as demonstrated by both RT-qPCR and *in situ* hybridization.

It could be speculated that PRV-1 in plasma is present as free viral particles or carried by extracellular vesicles, which are increasingly recognized for both intra- and inter-organism transmission for viruses [37,38]. Vesicle coating of rotaviruses are found to increase fecal-oral transmission [38]. Rotaviruses also belong to the family Reoviridae, and the fecal-oral route is considered a transmission pathway for PRV-1 [39] as well as for mammalian orthoreovirus [40]. If RNA viruses bud through cell membranes in extracellular vesicles, this could shelter the virus from antibody detection [41,42].

PRV-1 RNA levels in the persistent phase, as assessed by qPCR, were higher in kidney and spleen compared to blood and plasma. This was reflected by *in situ* staining of PRV in macrophage-like cells, in melano-macrophages, as well as in erythrocytes present in kidney and spleen. PRV-1 has been suggested earlier to infect macrophage-like cells in kidney and spleen based on a Canadian study of a field outbreak of HSMI [25]. Our data could indicate that PRV specifically target these long living immune cells, however, macrophage-like cells may stain because they actually phagocytize infected erythrocytes, as usually occurs in the spleen. Numerous macrophages and melano-macrophages are present at antigen trapping sites in kidney and spleen [36,43]. Melano-macrophages have earlier been demonstrated to be PRV target cells in Atlantic salmon [44]. Similar to all vertebrates, the survival, proliferation, differentiation, and functionality of bony fish macrophages are governed by macrophage-colony-stimulating factor, MCSF [45]. MCSF mediates its effects through a high affinity transmembrane receptor, the MCSF receptor (MCSFR) [46], which served as the macrophage identification marker in this study. The MCSFR expressed as reported both on myeloid precursors and derivative macrophage population in teleost fish [45], while other reports have claimed that MCSFR is specific to the macrophage population [47]. The MCSFR has been used to characterize monocytes/macrophages in Atlantic salmon [48], and stimulation with MCSFR has been found to enhance antimicrobial and phagocytic responses of macrophages in fish [45,49]. In mammals, MCSFR positive cells have been associated with an anti-inflammatory or immunosuppressive M2 macrophage population [50,51]. Duplex *in situ* hybridization demonstrated that some MCSFR positive cells in kidney and spleen are target cells for PRV-1. In an earlier study, using an antiserum against the non-structural viral protein µNS present only during viral replication [52], macrophage-like cells were associated with PRV replication [53]. Avian orthoreoreovirus (ARV), which also belongs to the Orthoreovirus genus, replicates in macrophages, and virulent ARV strains have shown enhanced ability to replicate in such cells [54].

Our study revealed that a number of additional so far un-characterized cell types, particularly in the erythropoietic tissue in the kidney, also stained positive for PRV-1. This encouraged us to use duplex *in situ* hybridization staining for the erythroid precursor cells together with PRV-1. Erythropoietin (EPO) plays a pivotal role in erythropoiesis by signaling through the specific erythropoietin receptor (EPOR) [55]. We found many EPOR positive cells in kidney stained positive also for PRV-1, suggesting there might be co-localization. This could indicate that the erythropoietic precursor cells were permissive to PRV-1, but future studies are required to elucidate this further. In fish, EPO is primarily produced in heart and transported to erythropoietic sites through blood [56], and triggers erythroid precursor cells to differentiate and proliferate into erythrocytes [57]. Despite of distinct differences between mammalian and teleost red blood cells, these functions are quite conserved [58]. Zebrafish (*Danio rerio*), have erythroid progenitor cells only in kidney [59], while in rainbow trout (*Oncorhynchus mykiss*) and European perch (*Perca fluviatilis*), spleen acts as additional organ for erythropoiesis [60]. In Atlantic salmon, an elevated level of EPOR expression in spleen suggests mobilization of early progenitors to cope with an anemic condition [61]. We found cells staining positive for PRV-1 in the tubular part of the kidney, but the dually stained EPOR and PRV-1 interstitial cells were primarily found in the hematopoietic centers. It could be speculated that these cells are involved in long-term infection of the host and contribute to generation of new PRV-1 infected erythrocytes.

Notably, *in situ* localization of PRV-1 RNA in different tissues illustrates a change in infection pattern when the infection moves into the persistent phase. Although high loads of PRV in heart is a hallmark of HSMI, PRV-1 levels in heart drops significantly during the persistent phase. On the other hand, viral RNA load is particularly high in kidney in the late persistent phase, indicating a shift in viral tissue tropism as the infection proceeds. This is in line with previous reports where tissue tropism was explored with qPCR methodology [4,12].

## 5. Conclusions

Taken together, this study describes the persistent phase of PRV-1 infection in Atlantic salmon and identifies cell types functioning as viral reservoirs. This work also identifies possible mechanisms of importance for PRV-1 persistence that can aid the understanding of PRV infection, possible viral recurrence and potential long-term effects of the infection.

## Figures and Tables

**Table 1:** Primer and probe sequences (5′-3′) for various PRV gene segments.

Target (PRV)	Primer/Probe	Concentration	Sequence (5′-3′)
L1	Fwd	400 nM	CGCACTCCCACAGATACAGTTC
	Rev		CGCGAGGTGTTACGTATTGTGA
M2	Fwd	400 nM	AGACTGGGAAGATCGTTGCTTT
	Rev		ATGCGTCTTGTTGAGTGTAGGT
M3	Fwd	400 nM	GGCCTGCATTGTGTCAACGT
	Rev		TGCGTTCAAGGTCGTCGTCA
S1 [12]	Fwd	400 nM	TGCGTCCTGCGTATGGCACC
	Rev		GGCTGGCATGCCCGAATAGCA
	Probe (FAM)	300 nM	ATCACAACGCCTACCT
S2	Fwd	400 nM	ATCAATGGCTTCGCTCTTCCTCTCTT
	Rev		TCTATATCCATTGCCGCATTTCCAGC
S3	Fwd	300 nM	AGCATCCTCACCATTTCCAAGCACTT
	Rev		AGAGGCACGATACACTAGAGCTTGA
EF1 α [24]	Fwd	300 nM	TGCCCCTCCAGGATGTCTAC
	Rev		CACGGCCCACAGGTACTG

**Table 2:** Target and control probes for *in situ* hybridization.

	Probe	Channel **	Accession no.	Target Region (bp)
Target	PRV-L3	C1	KY429945	415–1379
	MCSFR	C2	NM_001140235	434–1425
	EPOR	C2	NM_001140235	764–1754
Control	PPIB *	C1	NM_001140870	20–934
	DapB *	C1	EF191515	414–862
	DapB *	C2	EF191515	414–862

* Controls shown in Figure S3 ** Channel is a spectral attribution of the probes, which gives specific output with different amplification and detection systems.

## Supplementary Materials

The following are available online at https://www.mdpi.com/1999-4915/11/9/824/s1, Figure S1: Elongation Factor-1α expression, Figure S2: Positive and negative probes in singleplex in-situ hybridization assays, Figure S3: Positive and negative probes in duplex *in situ* hybridization assays. Figure S4: PRV localization in persistent phase. Figure S5: Negative control fish tissue for in situ hybridization. Table S1: RT-qPCR results from RNA isolated from blood cells, kidney and plasma with and without prior heating RNA at 95 °C for 5 min.
