# Supplementary material for: Erythroid Progenitor Cells in Atlantic Salmon (Salmo salar) May Be Persistently and Productively Infected with Piscine Orthoreovirus (PRV)
Source: Viruses. 2019 Sep 5;11(9):824. doi: 10.3390/v11090824 (PMC6784031; doi:10.3390/v11090824)
Supplement: Supplementary file 1 [file viruses-11-00824-s001.pdf]

Supporting information

Erythroid Progenitor Cells in Atlantic Salmon (*Salmo salar*) may be Persistently and Productively Infected with Piscine Orthoreovirus (PRV)

Muhammad Salman Malik [1], Håvard Bjørgen [2], Kannimuthu Dhamotharan [1], Øystein Wessel [1], Erling Olaf Koppang [2], Emiliano Di Cicco [3], Elisabeth F. Hansen [1], Maria K. Dahle [4] and Espen Rimstad [1,*]

1. Department of Food Safety and Infection Biology, Faculty of Veterinary Medicine, Norwegian University of Life Sciences, 0454 Oslo, Norway
2. Department of Basic Science and Aquatic Medicine, Faculty of Veterinary Medicine, Norwegian University of Life Sciences, 0454 Oslo, Norway
3. Pacific Biological Station, Fisheries and Oceans Canada, Nanaimo, BC V9T 6N7, Canada
4. Department of Fish Health, Norwegian Veterinary Institute, 0454 Oslo, Norway

* Correspondence: espen.rimstad@nmbu.no (ER); Tel.: +47-672-32-227

Table S1. RT-qPCR results (mean Ct values) from RNA isolated from (A) blood cells, (B) kidney and (C) plasma with and without prior heating RNA at 95°C for 5 min. wpc = weeks post challenge.

A

WPCs	Blood cells (Ct-values)				
	**Denatured**	**±SD**	**Not- Denatured**	**±SD**	**ΔCt**
3 wpc	21.20	3.95	22.17	3.65	-0.97
6 wpc	23.54	1.15	26.90	1.83	-3.36
9 wpc	25.60	2.96	29.35	3.05	-3.75
12 wpc	23.93	2.62	28.69	2.13	-4.76
15 wpc	25.44	2.87	30.47	2.07	-5.03
18 wpc	25.11	4.77	30.91	3.38	-5.8

B

WPCs	Kidney (Ct-values)				
	**Denatured**	**±SD**	**Not- Denatured**	**±SD**	**ΔCt**
3 wpc	21.29	4.15	24.76	-3.47	-3.47
6 wpc	17.40	1.36	23.24	-5.84	-5.84
9 wpc	21.24	2.27	27.40	-6.16	-6.16
12 wpc	20.49	1.50	26.34	-5.85	-5.85
15 wpc	21.79	0.90	27.75	-5.96	-5.96
18 wpc	21.74	2.58	27.95	-6.21	-6.21

C

WPCs	Plasma (Ct-values)				
	**Denatured**	**±SD**	**Not- Denatured**	**±SD**	**ΔCt**
3 wpc	22.13	3.71	31.10	4.22	-8.97
6 wpc	28.90	1.57	-	-	-
9 wpc	31.64	3.51	-	-	-
12 wpc	28.30	2.80	-	-	-
15 wpc	34.22	4.59	-	-	-
18 wpc	31.32	6.03	-	-	-

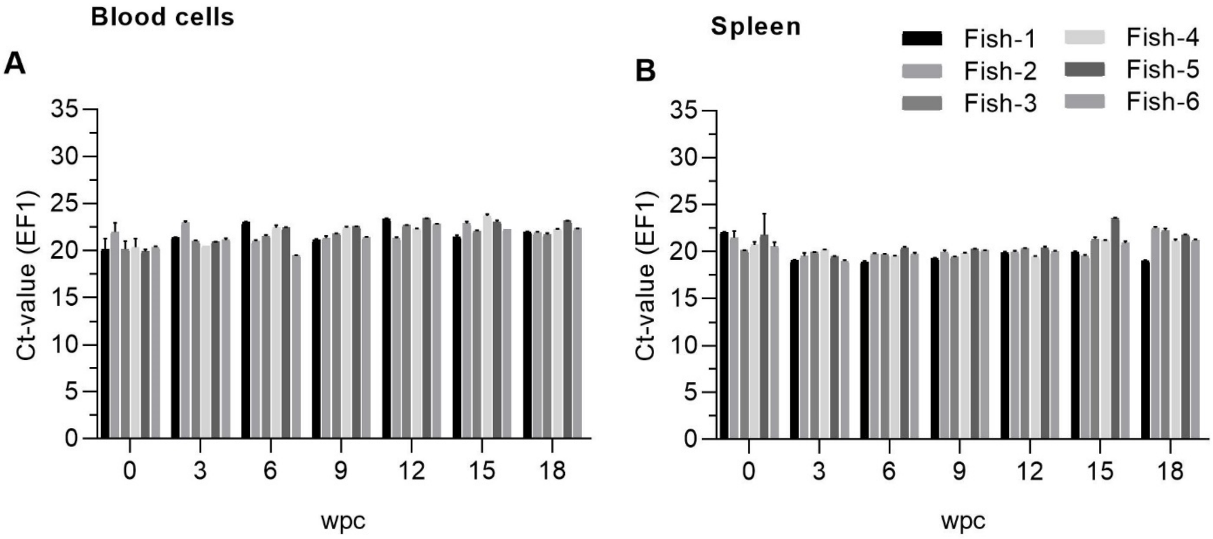

Figure S1. Elongation Factor-1α expression showing mean Ct values in (A) blood cells (B) spleen. Each bar represents a separate individual fish (n=6).

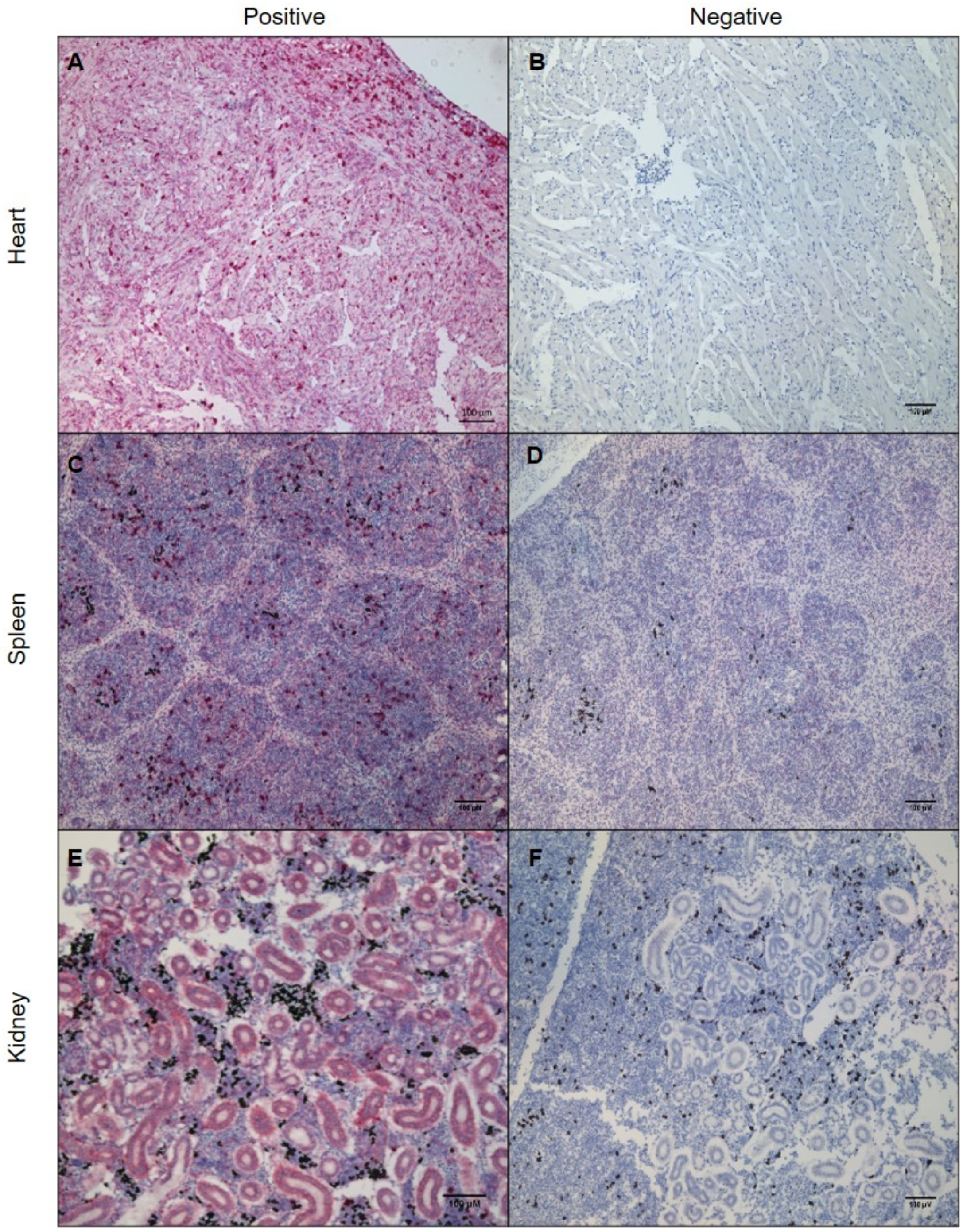

Figure S2. Positive and negative probes in singleplex *in-situ* hybridization assays. A, C, E show expression of probe designed for PPIB in Atlantic salmon heart (3 wpc), spleen (3 wpc) and kidney (6 wpc) as positive control (red). B, D, F: Samples from heart (3 wpc) spleen (3 wpc) and kidney (9 wpc) hybridized with probes designed for DapB related transcripts as negative control. (Scale Bar= 100 μm)

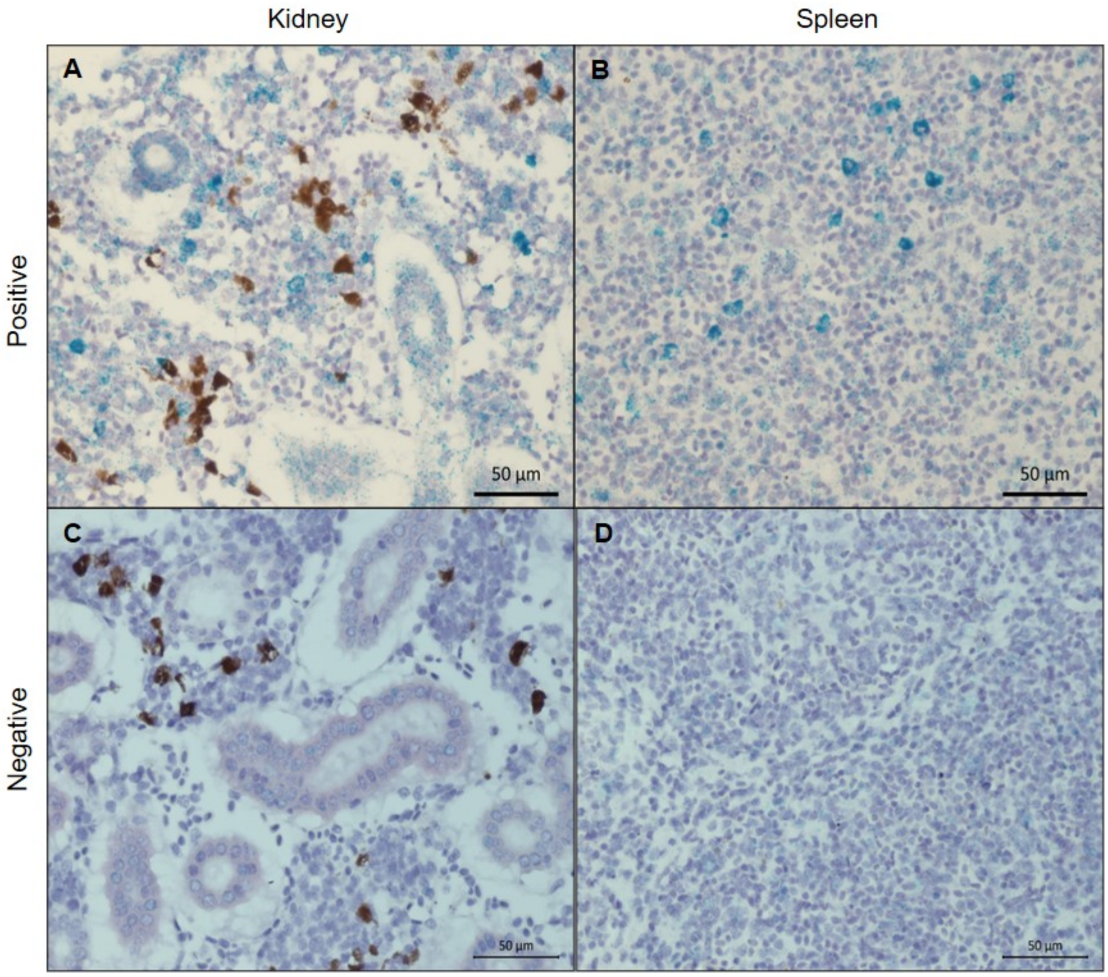

Figure S3. Positive and negative probes in Duplex *in-situ* hybridization assays. (A, B) showing expression of probe designed for PPIB (green) in A. salmon as positive control in kidney (12wpc) and spleen (12 wpc), respectively. (C, D) Samples hybridized with probes designed for DapB related transcripts as negative control in kidney (15 wpc) and spleen (15 wpc), respectively. (Scale Bar= 50 µm)

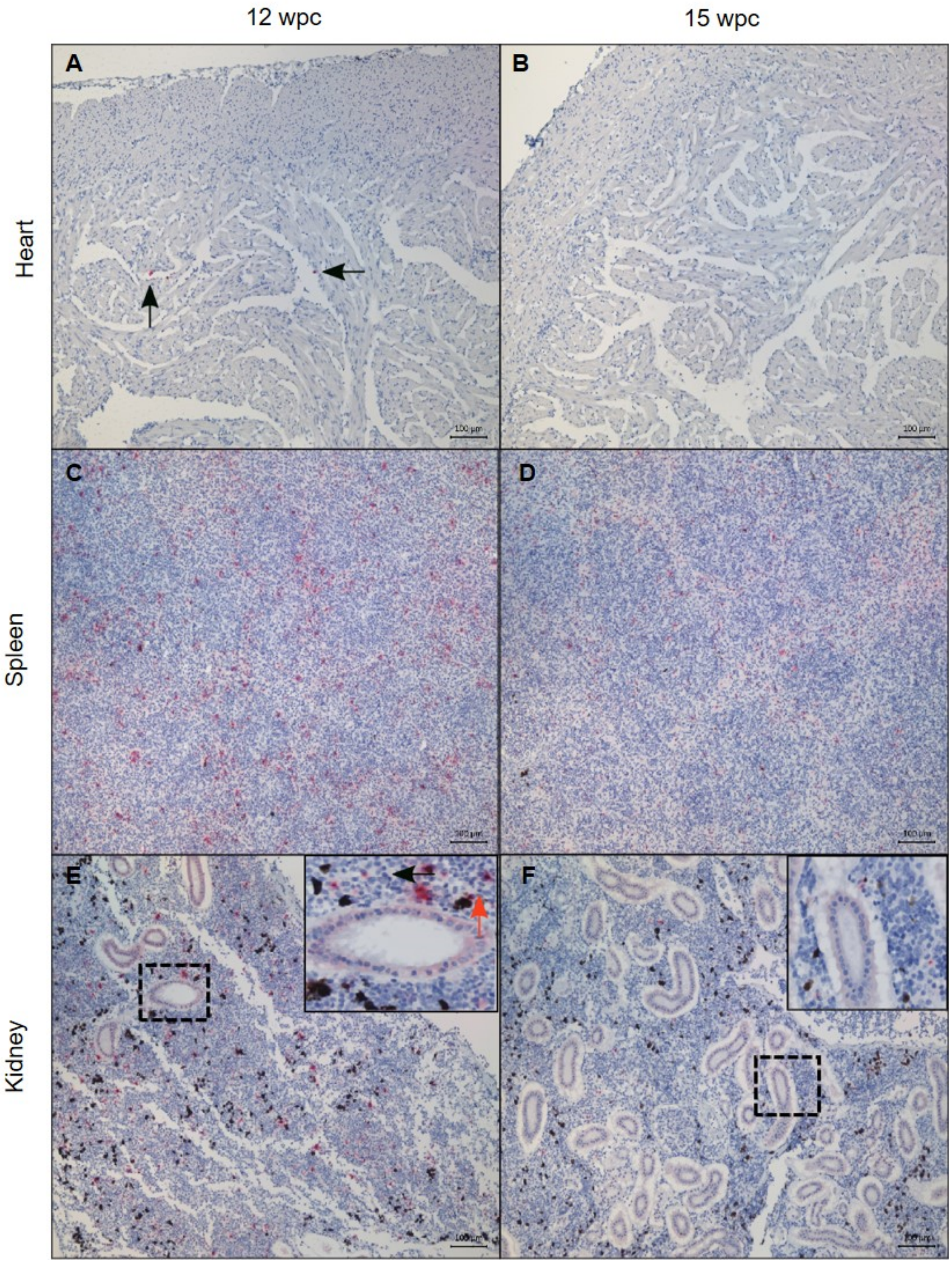

Figure S4. PRV localization in persistent phase Heart (A, B). (A) Few PRV positive (red) cardiomyocytes in spongy layer of ventricle at 12 wpc. (B) No PRV-1 positive cells detected at 15 wpc. Spleen (C,D). (C) A number of PRV positive RBCs and macrophage like cells present around red pulp area at 12 wpc, but (D) gradually clearing out at 15 wpc. Kidney (E, F) Significant number of positive cells present at peritubular region (black arrow) and other positive melano-macrophages both at 12 wpc and at 15 wpc (orange arrow and dotted rectangle). In situ hybridization using probes against PRV-1 L3 segment. Scale bar= 100 μm.

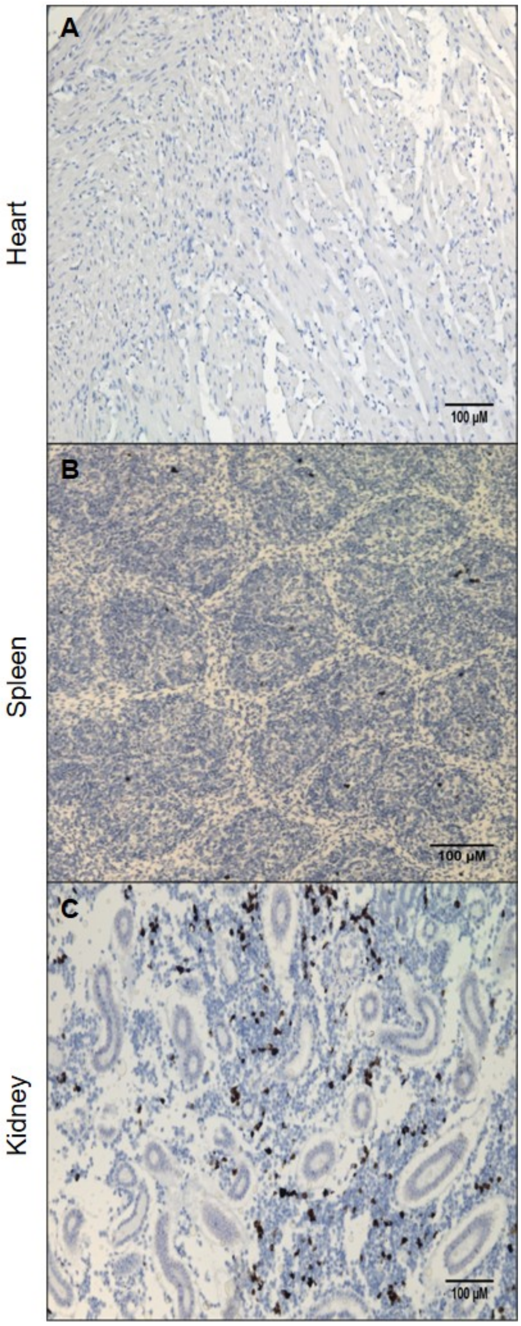

Figure S5. Control Fish tissues i.e heart, spleen and kidney (A, B & C) are negative for PRV1-L3 probe for *in situ* hybridization. Scale bar = 100 µm.
